# The effects of laser acupuncture on the modulation of cartilage extracellular matrix macromolecules in rats with adjuvant-induced arthritis

**DOI:** 10.1371/journal.pone.0211341

**Published:** 2019-03-18

**Authors:** Tien-Chien Pan, Yu-Hsin Tsai, Wen-Chi Chen, Yueh-Ling Hsieh

**Affiliations:** 1 Graduate Institute of Chinese Medicine, School of Chinese Medicine, College of Chinese Medicine, China Medical University, Taichung, Taiwan; 2 Departments of Urology, Obstetrics and Gynecology and Medical Research, China Medical University Hospital, Taichung, Taiwan; 3 Department of Physical Therapy, Graduate Institute of Rehabilitation Science, China Medical University, Taichung, Taiwan; Chang Gung University, TAIWAN

## Abstract

**Objectives:**

Articular cartilage damage related to irreversible physical disability affects most patients with chronic rheumatoid arthritis (RA). Strategies targeting the preservation of cartilage function are needed. Laser acupuncture (LA) can be an emerging alternative therapy for RA; however, its molecular mechanism underlying the beneficial effect on cartilage has not been elucidated. This study aimed to examine the potential chondroprotective effects of LA on extracellular matrix (ECM) macromolecules and proinflammatory cytokines in the articular cartilage of adjuvant-induced arthritis (AIA) rats and explore its related mechanisms.

**Design:**

Monoarthritis was induced in adult male Sprague–Dawley rats (250–300 g) via intraarticular injection of complete Freund’s adjuvant (CFA) into the tibiotarsal joint. Animals were treated with LA at BL60 and KI3 acupoints three days after CFA administration with a 780 nm GaAlAs laser at 15 J/cm^2^ daily for ten days. The main outcome measures including paw circumference, paw withdrawal threshold, histopathology and immunoassays of tumor necrosis factor-α (TNF-α), collagen type II (CoII), cartilage oligomeric matrix protein (COMP) were analyzed.

**Results:**

LA significantly reduced ankle edema and inflammation-induced hyperalgesia in AIA rats (P < 0.05). Moreover, the TNF-α levels were significantly decreased while CoII, COMP and proteoglycans proteins were significantly enhanced following LA stimulation of the AIA cartilage compared to those treated with sham-LA (P < 0.05).

**Conclusions:**

LA attenuates cartilage degradation in AIA rat by suppressing TNF-α activation and up-regulating ECM macromolecules, suggesting LA might be of potential clinical interest in RA treatment.

## Introduction

Rheumatoid arthritis (RA) is characterized by persistent synovial inflammation and hyperplasia, which eventually lead to articular cartilage destruction and disability [1]. Articular cartilage damage is related to irreversible physical disability of RA and special attention should be given to therapeutic interference with cartilage damage [2]. However, most studies on the treatment strategies of RA focused on reducing synovial hyperplasia and inflammatory cell infiltration, with less attention being paid to the delay in the pathogenesis of cartilage damage.

Chondrocytes of articular cartilage are embedded in a matrix comprising water, type II collagen (CoII) and proteoglycans which provide tensile and cushioning properties of articular cartilage, respectively [3]. Cartilage oligomeric matrix protein/thrombospondin-5 (COMP/TSP5) is an abundant cartilage extracellular matrix (ECM) protein produced by the cartilage as an original molecular marker of cartilage [4]. It can interact with major cartilage ECM components and collagens to maintain cartilage integrity and regulate chondrocyte function and cell death [5]. It is also known tumor necrosis factor-alpha (TNF-α) in synovial fluid plays an essential role in the inflammatory pathogenesis of RA. Therefore, in RA, many pathological factors including proinflammatory cytokine TNF-α overexpression, degradation of proteoglycan, breakdown of the collagen network and several other factors result in an imbalance between anabolic and catabolic processes of extracellular matrix (ECM), finally inducing cartilage destruction [6].

Acupuncture providing real and meaningful benefits for alleviating pain and improving physical function has been widely used as an alternative therapy in patients with arthritis through either needle or laser acupuncture (LA)[5,7,8]. The analgesic effect of LA on chronic pain has been proven by photobiomodulation through acupuncture points and it is an alternative to low-level laser therapy. Among all the proposed mechanisms by which acupuncture works, the anti-inflammatory effect has been the most often mentioned and is supposed to provide nonanalgesic effects via suppression of the inflammatory response, improved blood flow, or relaxation of muscle tone, but they are still largely conjectural [7, 9]. The detailed molecular mechanisms underlying the chondroprotective effects of LA in RA are not fully understood.

This study hypothesizes low-level laser therapy stimulating selected acupuncture points, LA, may have a beneficial biological effect on the protection of inflammation-induced cartilage defects. Therefore, we attempted to study the chondroprotective effects of LA on cartilage protection by using COMP, CoII and TNGF-α immunoassays through an *in vivo* model of adjuvant-induced arthritis (AIA) rats.

## Materials and methods

### General design

**Fig 1:**
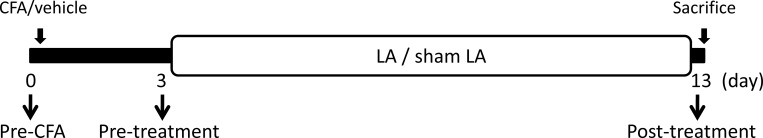
Experimental design. Complete Freund’s adjuvant (CFA) or vehicle was intraarticularly injected at Day 0. Pain withdrawal threshold and swelling assessments were examined at timepoints of Pre-CFA, Pre-treatment and Post-treatment. Three days after injection of CFA (Day 3), animals were treated with laser acupuncture (LA) and sham-operated LA (sLA). Following the 10-day treatment (Day 13), animals were sacrificed for histology and immunohistochemistry assays.

Monoarthritis was induced at a unilateral ankle joint in all animals by intra-articular injection of Complete Freund’s adjuvant (CFA). After three day of CFA induction, the arthritic animals (n = 30) were randomly divided into two groups according to laser irradiation treatments named: LA group (n = 15): animals treated with laser irradiation (15 J/cm^2^) at the acupuncture points and the sLA group (n = 15), animals treated with sham-operation laser irradiation (0 J/cm^2^) at the acupuncture points. The ankle contralateral to the CFA-injection side was the internal control for vehicle injection. The treatments using laser or sham irradiation were given on the 3rd day after CFA induction for ten consecutive days. The evaluation instruments were edematous swelling of the paw, pain behavioral assessments, histology, and immunohistochemistry. Behavioral assessments were performed before CFA (pre-CFA, Day 0), before (pre-treatment, Day 3) and after (post-treatment, Day13) the 10-day LA treatment (1 session/day). After completing the treatments, the animals were sacrificed for histopathology assessment and immunoassays. The flow diagram is presented in Fig 1.

### Animal preparation

Thirty adult male Sprague-Dawley (CD^®^(SD) IGS BR; purchased from BioLASCO Taiwan Co., Ltd.) rats weighing 250–300 g were kept in the Laboratory Animal Center of China Medical University. Effort was made to minimize discomfort and to reduce the number of animals used. All animal experiments in this study were conducted with the procedure approved by the Institutional Animal Care and Use Committee (IACUC) of China Medical University in accordance with the Guidelines for Animal Experimentation (No 100-30-N).

### Induction of monoarthritis by ultrasound-guided CFA injection

Monoarthritis was induced by an injection of CFA into the unilateral ankle articular cavity selected randomly. The rats were briefly anesthetized with 4% isoflurane (AERRANE, Baxer Healthcare Corporation, Puerto Rico). Ultrasound (Terason t3000 Ultrasound System, Terason Division, Teratech Corporation, MA, USA) guided injection was performed on the lateral side of tibiotarsal joint, with the transducer in the sagittal plane showing the distal end of the tibia and proximal part of the tarsus in the image plane. A 28-gauge needle was vertically inserted distally into the articular cavity from the gap between the tibiofibular and tarsus bone. CFA (10 mg mycobacterium, F5881, Sigma, MO) / saline (as a vehicle control) at a volume of 50 μl was then injected into one side individually. After induction, animals were separately placed in clear acrylic containers (10½" W × 19" D × 8" H), allowing free movement for at least 24 h to let them adjust to these conditions before any experimentation was performed.

### Laser acupuncture

Under slight anesthesia, the animals were treated with LA at the BL60 (Kunlun, between the tip of the external malleolus and the Achilles tendon) and KI3 (Taixi, between the tip of the medial malleolus and the attachment of the Achilles tendon) acupoints daily for 10 days. A continuous 780 nm GaAlAs diode laser (Aculas-Am series, Multi-channel laser system; Konftec Corporation, Taipei, Taiwan) was used in the treatment. Laser irradiation was utilized with a spot size of approximately 0.2 cm^2^ at fluence at 50 mW per session for 60 sec per spot (energy density: 15 J/cm^2^). The output of the equipment was routinely checked using a Laser Check Power Meter (Coherent Inc, Santa Clara, CA, USA). A similar procedure was applied to the sLA group using sham irradiation with the power set at 0.

### Edematous swelling and pain threshold assessments

The extent of peripheral swelling was assessed by measuring the circumference of the paw at the vehicle- and CFA-injected sites with a flexible tape. The paw circumference was obtained by averaging three measurements. The pain thresholds were determined by nociceptive thresholds to mechanical stimulation. The test consisted of evoking a hind paw flexion reflex with a handheld force transducer (electronic von Frey anesthesiometer, IITC Inc., CA, USA) adapted with a 0.5 mm^2^ polypropylene tip. In a quiet room, the rats were placed in acrylic cages (32 × 22 × 27 cm high) with a wire grid floor for 15 -30 min habituation prior to testing. The test consisted of poking a hind paw to provoke a flexion reflex followed by a clear flinch response following paw withdrawal. The filaments were applied with a gradual increase in pressure until a withdrawal reflex response was finally detected from the animal as the paw withdrawal threshold (g). The intensity of the pressure was recorded and the final value for the response was obtained by averaging five measurements. All assessments including paw withdrawal and swelling measurements were performed with the assessor blinded with respect to treatment.

### Histopathological examination

Animals were euthanized by anesthetic overdose after treatments and then their hind ankles were collected for histological, immunohistochemical and immunofluorescent analyses. The specimens were fixed in 4% phosphate buffered saline (PBS)-buffered paraformaldehyde for 48 hours and decalcified in PBS-buffered 10% ethylenediaminetetraacetic acid (EDTA) for 20 days at 4°C. Specimens were frozen with liquid nitrogen immediately after decalcification and kept at -80°C until analysis. The ankle joint tissues were frozen and sagittal sections of 4 μm thickness were cut serially with a freezing microtome. Each specimen produced approximately 60 sections. Each staining assay was examined in 10 alternate sections per ankle joint per rat which were selected by a systematic-random series with a random start for analysis. The frozen sections were first mounted on poly L-lysine (Sigma, P8920)-coated slides. Hematoxylin and eosin (HE) and Safranin-O (S2255-25G, Sigma) / Fast green (FCF-Bio Basic Inc. FB0452)-stained sections were used to assess the structures, cells, pannus formation and loss of proteoglycan, respectively. The variants of rheumatoid synovitis were analyzed under light microscopy (BX60; Olympus, Tokyo, Japan) with particular attention being paid to the inflammatory cells infiltrating density.

### Immunohistochemical and immunofluorescent analyses

The frozen sections were treated with proteinase K (Sigma, St. Louis, Mo, USA) at 0.1 mg/mL for 20 min at room temperature to unmask epitopes followed by a PBS rinse. Sections were incubated with blocking buffer (Power Block, Biogenex, USA) for 2 h at room temperature followed by incubation overnight at 4°C with the rabbit polyclonal anti-Collagen II antibody (anti-CoII, 1:50, ab79013, Abcam, Cambridge, UK), rabbit polyclonal anti-TNF-α antibody (1:200, ab 6671, Abcam, Cambridge, UK), or mouse monoclonal anti-thrombospondin 5 antibody (designated TSP 5, cartilage oligomeric matrix protein, or COMP) (1:50, sc-33696, Santa Cruz Biotechnology, Inc. Cambridge, UK). After three washes with PBS containing 0.05% Tween-20 for 10 min, sections were incubated with the secondary antibody. The sections with anti-CoII were incubated with biotinylated anti-rabbit IgG (Jackson Immunoresearch, PA, USA), followed by a peroxidase-conjugated streptavidin incubation (Jackson Immunoresearch, PA, USA). The sections were visualized as brown precipitates by 3,3′-diaminobenzidine (DAB, Pierce, Rockford, IL, USA) as a substrate. The sections with anti-TNF-α and anti-COMP were incubated with Alexa Fluor 594-conjugated goat anti-rabbit IgG (1:500, AS039, ABclonal Inc., MA, USA) and Alexa Fluor 488-conjugated goat anti-mouse IgG (1:500, AS037, ABclonal Inc., MA, USA) in the dark for 2 hours, respectively. Finally, the fluorescent-labeled sections were counterstained with 4′,6-diamidino-2-phenylindole (DAPI, Life Technologies, Carlsbad, CA) for 5 min and the nucleus position was fluoresced by blue light at 340–380 nm. Normal mouse and rabbit IgGs (Jackson Immunoresearch, PA, USA) were used to replace the primary antibodies as negative controls.

### Quantitative analysis

The slides were examined and photographed at five randomly selected fields at 200× magnification using a light microscope (BX43, Olympus America Inc. NY, USA) and a cooled digital color camera with a resolution of 1360 × 1024 pixels (DP70, Olympus America Inc. NY, USA). The digital images were analyzed using computer-based morphometry, Image-Pro Plus 4.5 software (Media Cybernetics, Silver Spring, USA). Based on the automatically calculated parameters, the labeled area by Safranin-O-, DAB- or Alexa-positive staining cells in each section was measured. The percentage of positive reactivity pixels to total pixels in the cartilage (%) was analyzed. All counts were performed by at least two independent individuals in a blinded manner.

### Statistical analysis

The results of pain withdrawal threshold and ankle circumference expressed as mean ± standard error mean (SEM) for comparing the serial changes within a group were analyzed using repeated measures ANOVA. A paired *t*-test was conducted to compare the mean differences between timepoints within a group in both CFA-injected (arthritic) side and vehicle control side. All data in histopathology and immunoassays are expressed as mean ± standard deviation (SD). One-way ANOVA was used to determine the differences in the levels of CoII, TNF-α- and COMP-like immunoreactivities among vehicle controls, sLA and LA groups. Scheffe’s method was used to examine post hoc for the comparisons between groups. A P value of < 0.05 was considered statistically significant. All data was analyzed using SPSS version 20.0 for Windows (IBM SPSS Statistics, USA). The data have been deposited in the figshare database (DOI: https://figshare.com/s/4c675d8af11b77c99d11).

## Results

### Effects of LA on CFA-induced edema

**Fig 2:**
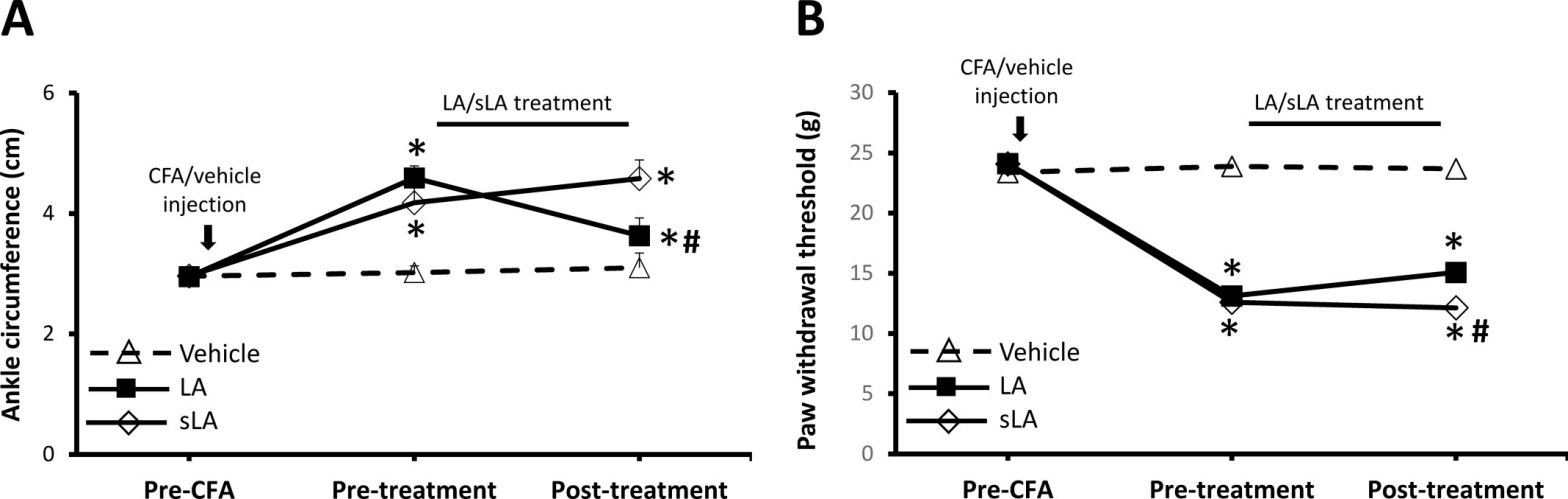
**Development of ankle circumference (A) and pain withdrawal threshold (B) in vehicle injection, CFA-induced arthritic rats treated with laser acupuncture (LA) and sham-operated laser acupuncture (sLA) at timepoints of before CFA injection (Pre-CFA), before and after treatments (Pre-treatment and Post-treatment)**. Values are means ± SD. * indicates a significant change in the parameter assessed (P < 0.05), when values are compared with vehicle control values. # indicates a significant change in the parameter assessed (P < 0.05), when values are compared with sLA values.

The serial alterations of the circumference of the ankle (mean ± SEM) throughout the entire experiment for each group are shown in Fig 2A. Following CFA-induction, all animals developed severe edema in the injected paw. There were no significant differences in the non-injected intact paw of the vehicle control side in terms of circumference among the timepoints of pre-CFA and pre-treatment, and post-treatment conditions (repeated measures ANOVA, df = 2, F = 2.873, P *>* 0.05). The circumference of the CFA-injected paw gradually increased, reaching a mean edema of 50.91% ± 2.94%, whereas there were significant differences in edema between the pre-CFA and pre-treatment conditions (paired *t*-test, P *<* 0.001). Following LA treatment, significant decreases in circumference of arthritic paws were observed in the LA group compared to those of pre-treatment (paired *t*-test, P *<* 0.001). However, there was no difference in the edema of the arthritic paws in the sLA group between the timepoints of pre- and post-treatment (paired *t*-test, P = 0.142).

### Effects of LA on CFA-induced inflammatory mechanical nociception

The serial alterations of the paw withdrawal threshold (mean ± SEM) over the course of the entire experiment for each group are shown in Fig 2B. The mean threshold was 23.87 ± 0.27 g in the pre-CFA condition. However, following CFA-induction, it decreased to 12.85 ± 0.22 g at the pre-treatment condition. There was a significant difference from the pre-CFA condition (paired *t*-test, P *<* 0.001). The threshold was significantly higher in the LA group at the post-treatment condition than those of pre-treatment (paired *t*-test, P = 0.044), however, no significant difference was observed between the pre-treatment and post-treatment conditions in the sLA group (paired *t*-test, P = 0.441). At the post-treatment condition, there was a significantly lower threshold found in the LA-treated side than those in the vehicle-treated side (Scheffe’s method, P < 0.001), but a significantly higher threshold existed after LA treatment than those treated with sLA (Scheffe’s method, P = 0.007).

### Effects of LA on CFA-induced histopathological changes in the cartilage and synovium

**Fig 3:**
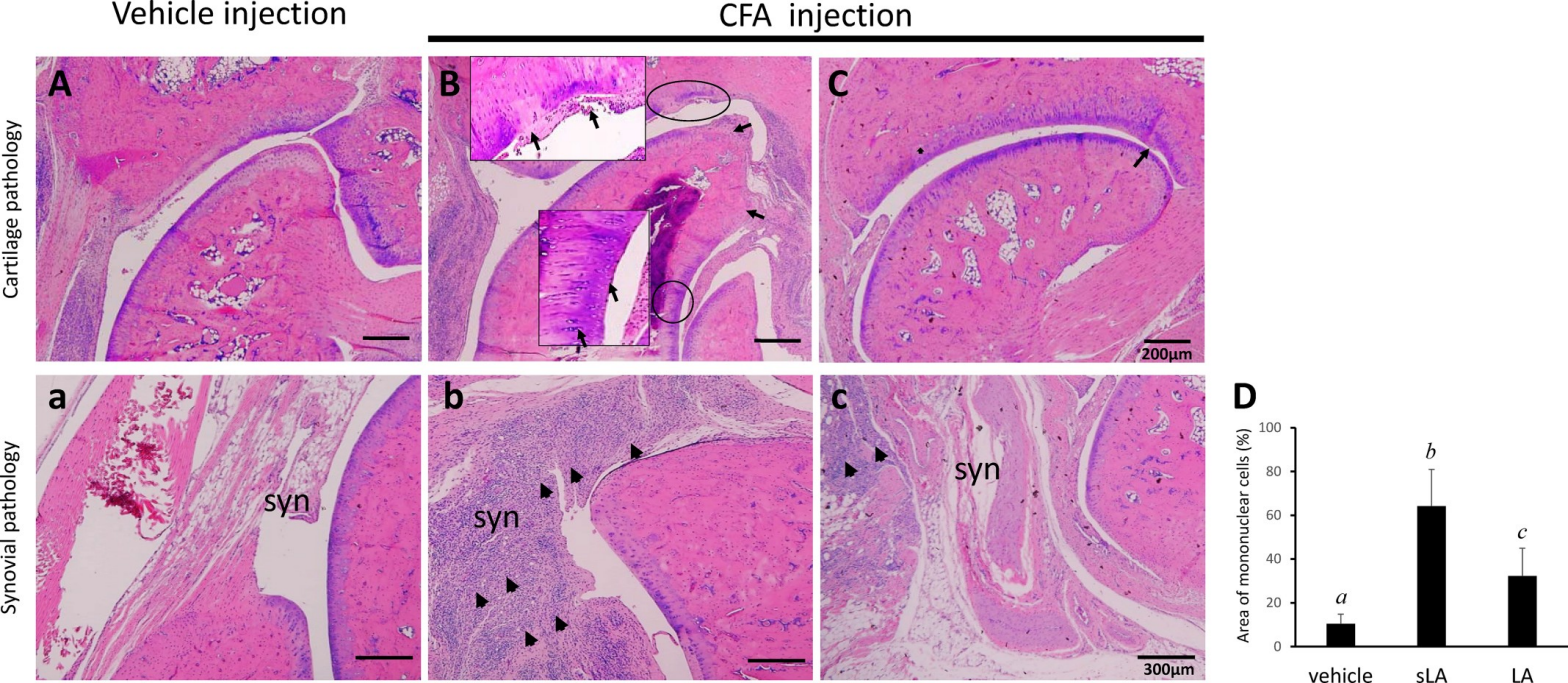
**Representative HE sections of the hind paws obtained from vehicle (A) and CFA-induced arthritic animals treated with sham-operated and laser acupuncture (sLA and LA, B and C).** Histology showed articular cartilage changes (A-C) and synovial (syn) inflammation (a-c) in the CFA-injected side of sLA and LA groups when compared to the vehicle side. The articular cartilage surface was smooth and evenly stained on the vehicle control side of each group, but there were apparent articular cartilage fissures, extending from the surface to the depth with significant loss of staining in the sLA group. Synovium showed thickening and widening of the synovial membrane and infiltration of inflammatory cells being less in rats from the LA groups (c) and apparent in rats of sLA group (b). The bar graph shows a statistical difference in the area of infiltrating inflammatory cells among the ankles treated with vehicle, sLA, and LA (D). Each value represents the mean ± SD. Values in each bar with different italic letters (*a*,*b*,*c*) indicate significant difference at confidence level of P *<* 0.05 tested by the Scheffé post hoc test. The arrows indicate areas of cartilage disruption and chondrocyte disorientation with clusters of chondrocytes. The arrowheads indicate areas of inflammatory cell infiltration in synovium.

Co-occurrence of synovial inflammation and cartilage erosion was found in this CFA-induction RA rat model (Fig 3). Observations of the cartilage surface and cell alignment using HE staining showed the cartilage in the vehicle-treated side was smooth, while cartilage lesions were apparent in the CFA-treated groups (Fig 3A). The sLA group exhibited degradation changes, with decreased cartilage thickness, damaged collagen fibers, and chondrocyte disorientation with clusters of chondrocytes in the superficial and middle zones of the articular cartilage (Fig 3B). Surface congruity and integration of the lesion to the adjacent articular cartilage were better in the LA group (Fig 3C).

Less hyperplasia and hypertrophy of the synovium with pannus and vessel formation and infiltration of mononuclear cells were seen on the vehicle-injection side (Fig 3A) than the CFA-injection side of the LA and sLA groups (Fig 3B and 3C). There were significant differences in the infiltration of mononuclear cells analyzed by HE staining observed between the ankle joint synovium from the vehicle- and CFA-injection sides (paired *t*-test, P < 0.001, data not shown). Thickening and widening of the synovial membrane and infiltration of the inflammatory cells were apparent in rats from the sLA group (Fig 3B). Conversely, synovium from the LA group showed they were less inflamed, as revealed by the decreased number of inflammatory cells and hyperplasia in the synovial membrane (Fig 3C).

### Effects of LA on the modulation of proteoglycans, COII, COMP and TNF-α expression in cartilage following CFA-induction

**Fig 4:**
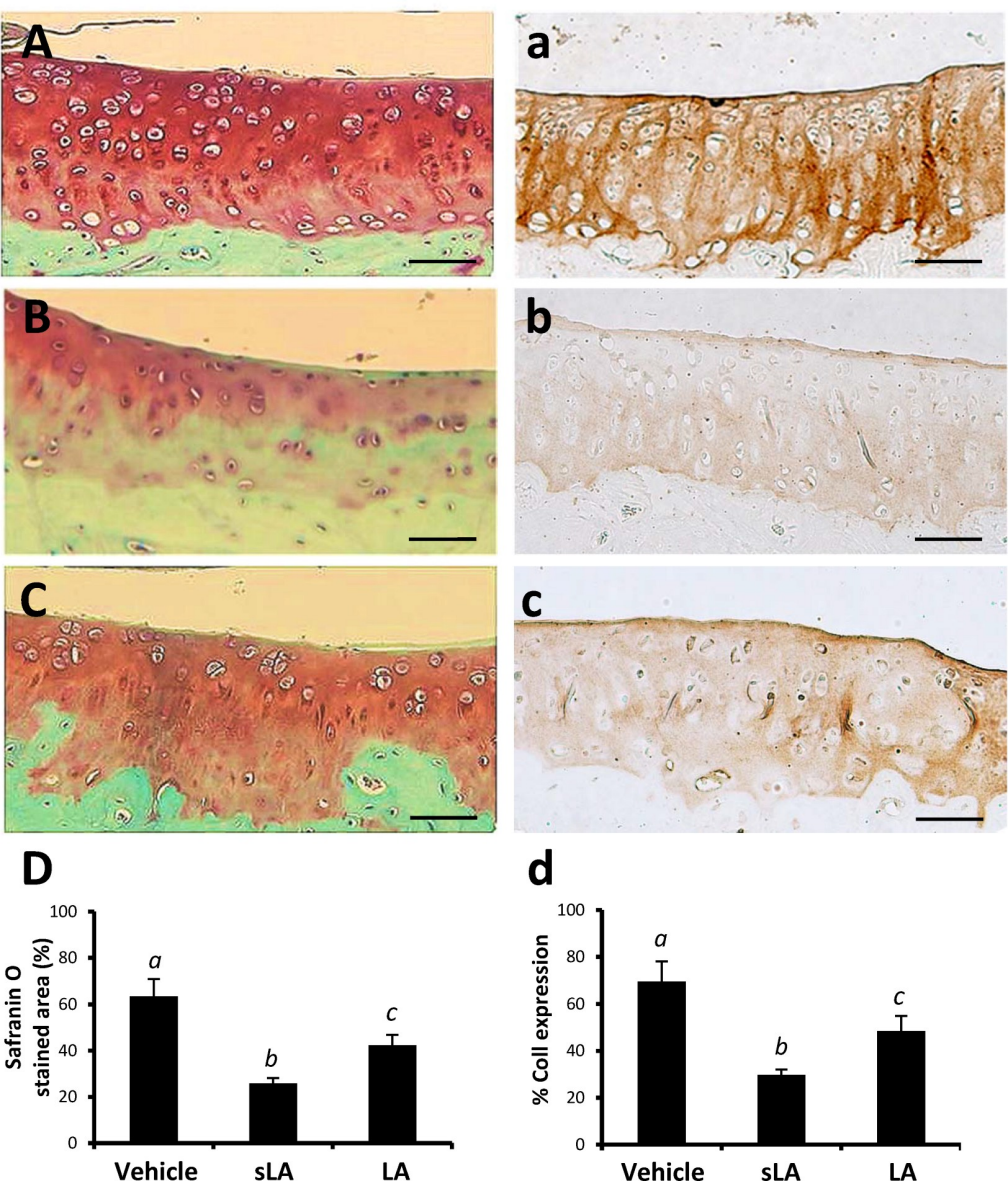
**Representative images of surface cartilage damage at the tibial plateau with collagen type II (CoII) immunoreactivity and safranin O/Fast Green staining in vehicle control (A and a), CFA combined with sham-operation (sLA, Band b) and laser acupuncture (LA, C and c) (sLA and LA) groups, respectively.** At higher-power magnification, it is evident that marked increase of positive collagen type II immunoreactivities (brown staining) and proteoglycan safranin O (dark orange) were clearly localized in the LA group compared with sLA. Quantitative analysis of positive-labeled cells in cartilage for safranin O (D) and collagen type II immunohistochemistry (d) in each group was presented in mean ± SD. Different italic letters (*a*,*b*,*c*) indicate significant differences between groups using Scheffe’s post hoc test (P *<* 0.05). A scale bar indicates 50 μm.

The extracellular matrix was intensely Safranin-O stained on the vehicle-treated side (Fig 4A). Matrix Safranin-O staining intensities in the CFA-treated sides of sLA (Fig 4B) and LA (Fig 4C) groups were gradually decreased from the articular surface and pericellular area. The Safranin-O positive area was significantly higher in the LA group than those in the sLA group (Fig 4D, Scheffe’s method, P = 0.014).

CoII immunohistochemistry analysis of the cartilage from the vehicle-treated side and CFA-injection side of the sLA and LA groups are shown in Fig 4. The CoII-like immunoreactivity of the vehicle-treated sides was relatively normal compared with that of the surrounding tissue, indicating much more type II collagen protein was formed (Fig 4E). The protein levels of CoII were markedly lower in the sLA group than in the LA group (Scheffe’s method, P < 0.001, Fig 4F and 4G). Although the LA group enhanced the CFA-induced reduction of CoII expression in cartilage, its protein level was also lower than those of the vehicle control (Scheffe’s method, P *<* 0.001, Fig 4H).

**Fig 5:**
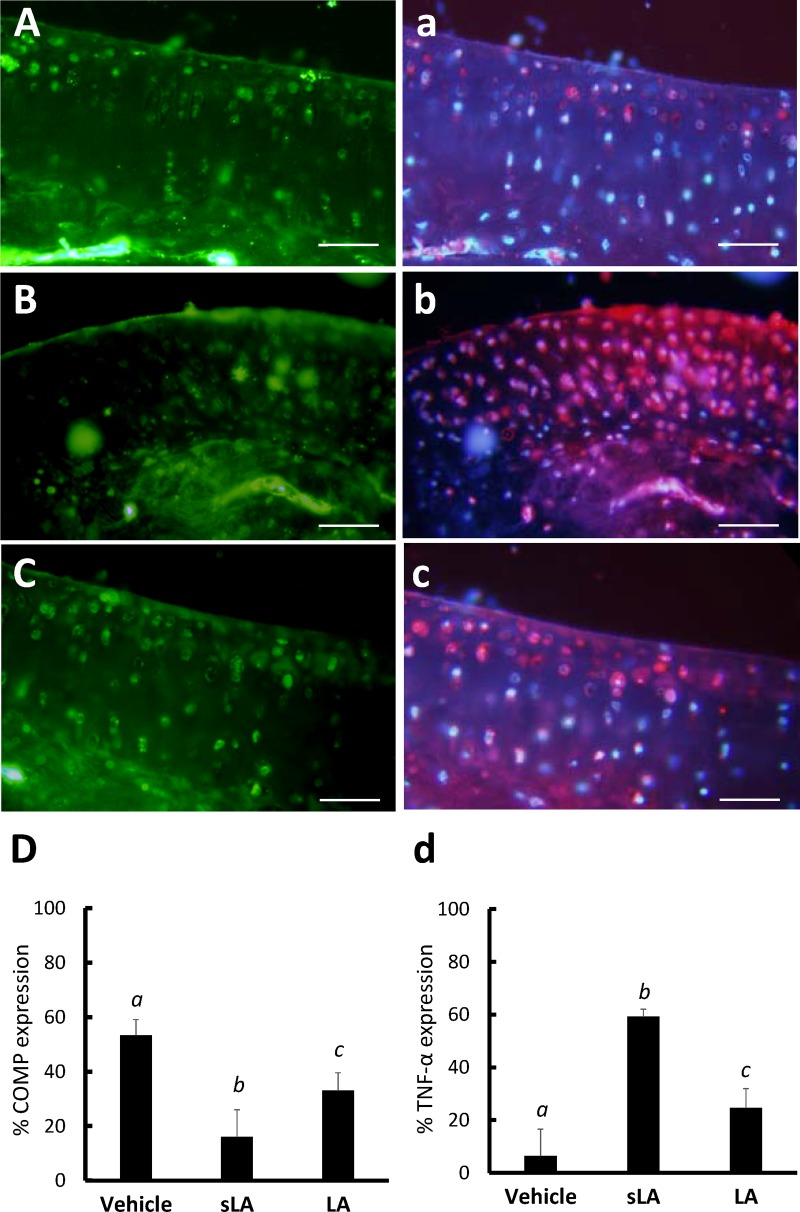
**The immunofluorescent expressions of COMP (Alexa Fluor 488-green) and TNF-α (Alexa Fluor 594-red) immunoreactivity in the vehicle control (A and a), CFA combined with sham-operation (sLA, B and b) and laser acupuncture (LA, C and c) groups, respectively.** The level of COMP- and TNF-α-like immunoreactivity were counted in five randomly selected high-power fields (×200) in each section (D and d). The bar graph shows a statistical difference in the COMP- and TNF-α-like immunoreactive cells among the vehicle control, sLA and LA groups. A decrease in COMP expression and elevation in TNF-α expression in the cartilage from the sLA group were noted compared with the vehicle control and LA groups. Each value represents the mean ± SD. Different italic letters indicate significant differences between groups using Scheffe’s post hoc test (P < 0.05). A scale bar indicates 50 μm.

Immunofluorescent staining revealed the intensity of COMP-like immunoreactivity on the vehicle-treated side (Fig 5A) was higher compared to the CFA-treated side of the sLA (Fig 5B) and LA (Fig 5C) groups (Scheffe’s method, P < 0.001). LA significantly enhanced the ECM with COMP-like immunoreactivity in the cartilage compared with the sLA group (Scheffe’s method, P < 0.001, Fig 5D).

Significantly lower levels of TNF-α were found on the vehicle-treated side using immunofluorescent staining (Fig 5A). It also revealed more abundant expressions of TNF-α-like immunoreactivities in the sLA group (Fig 5B) compared with those in the LA group (Fig 5C). A statistically significant TNF-α cytokine reduction was observed in the LA groups compared with the sLA (Scheffe’s method, P < 0.001, Fig 5D). However, a significant difference was also found between the LA and vehicle controls (Scheffe’s method, P < 0.001, Fig 5D).

## Discussion

Our study results demonstrated the chondroprotective effects of LA in reducing proinflammatory cytokine TNF-α, and enhancing the protein levels of proteoglycans, CoII and COMP in the superficial and transitional zones of the cartilage, indicating improvement of joint inflammation and cartilage ECM anabolic bioactivity in AIA rats. LA is also a promising treatment modality to elevate the nociceptive withdrawal threshold and reduce edema. Therefore, LA appears to promote cartilage repair in inflammation-induced cartilage destruction, probably due to its efficiency in modulating most of the main factors involved in the pathogenesis of this disease.

Cartilage is a special avascular and aneural tissue with sparse chondrocytes embedding in the dense ECM. The cartilage ECM macromolecules, collagens and proteoglycans play a central role in cartilage functionality, primarily in establishing the mechanical functions [5]. Activation of CoII can obviously promote the growth of cartilage cells, and promote regeneration of injured cartilage cells in the surrounding environment. Proteoglycans are important to retain collagen in the matrix when a new matrix is produced [10]. Therefore, CoII and proteoglycan have always been used as the criteria for identifying and evaluating the chondrogenic capacity of cells [11,12][However, the quantity of these ECM macromolecules is currently an obvious obstacle for their inadequate production, even though regeneration of the cartilage lesions involves the use of engineered constructs [11]. Histopathological change in synovial and articular cartilage with inflammation and ECM degradation are widely regarded as features of the progression of cartilage degradation in AIA models of RA [13,14]). Decreased CoII protein levels and safranin-O-staining intensity provide direct evidence for the rapid development of degenerative changes in the cartilage of RA [15,16]. Therefore, strategies targeting the promotion of CoII and proteoglycan production and accumulation in damaged cartilage tissues need to be stressed. This study demonstrated the protein levels of CoII and proteoglycans were enhanced following LA stimulation in AIA animal’s ankle cartilage, indicating preservation of ECM properties in AIA cartilage.

COMP is a structural molecule in the cartilage which may be able to regulate cellular activities and respond to the environment in the surrounding cartilage matrix by mediating chondrocyte attachment through interactions with several integrins (4). In adult articular cartilage, COMP immunoreactivity can be expressed in the cartilage at chondrocytes in the pericellular matrix, territorial and interterritorial matrix [4,17]. Regulation of chondrocytes is a key problem for protection against cartilage degradation and if cartilage destruction exists, COMP may play an important role in regulating the differentiation and function of chondrocytes[18]. Moreover, production of proinflammatory cytokines, TNF-αcauses the release of proteolytic enzymes, matrix metalloproteinases, and degradation of the connective tissue of the joint. With the advent of targeted biological treatments, anti-TNF therapy has been revolutionized for managing RA[19]. In this study, AIA reduced COMP protein expression and induced TNF-α overexpression in cartilage, but LA significantly enhanced COMP-like immunoreactivity and reduced TNF-αoverexpression in the cartilage compared to sLA treatment. Therefore, LA may have a potentially beneficial effect on protection against cartilage degradation by modulating the COMP and TNF-α proteins in AIA rats.

A basic study proved the therapeutic effect of acupuncture on AIA-induced RA via decreasing the pro-inflammatory cytokines of Interleukin (IL)-1, IL-6 and TNF-α and increasing the antiinflammatory cytokines of IL-4 and IL-10 in RA serum and joints [7]. A previous study using electroacupuncture on the ST36 (Zusanli) and BL60 (Kunlun) acupoints of AIA rats demonstrated the molecular mechanism of acupuncture of RA was found by reducing the expression of toll-like receptor 4, myeloid differentiation factor 88 (MYD88), and NF-κB which play an important role in treating adjuvant arthritis[20]. Electroacupuncture at acupoints ST-36, GB-39 and BL-23 (Zusanli, Xuanzhong, and Shenshu) also reduced the expression levels of NF-κB and TNF-α in the synovial tissues of AIA rats[21]. However, the specific mechanisms through which LA reduces AIA inflammation-induced cartilage destruction in RA are still not fully understood. Therefore, in accordance with this previous study [21], our results indicated the restoration of cartilage with LA, which can enhance the expression of CoII, proteoglycans and COMP and reduce the TNF-α in cartilage, probably because LA stimulated photomodulation action promotes chondrocyte activity.

Based on the widespread application of acupuncture in various diseases, it is well known analgesia modulation is commonly thought to be the main subject of acupuncture [22]. In general, analgesia was thought to be obtained by short-term acupuncture via central pathways by secreting a number of bioactive chemicals, such as spinal opioids and serotonin [23,24]. In several clinical trials, acupuncture can significantly reduce pain, morning stiffness, tender joint counts, swollen joint counts and improve the quality of life of patients with RA [7, 25]. Previous studies reported LA stimulating the acupoints ST36 (Zu San Li) and TH5 (Waiguan) was effective in reducing edema and acute inflammatory pain in AIA rats and spontaneous pain and thermal hyperalgesia in neuropathic pain rats[26]. In our study, LA significantly reduced ankle edema and inflammation-induced hyperalgesia in AIA rats, which is consistent with the results of previous research, indicating the analgesic effects of LA in the AIA model.

Moreover, the modulation of the immune system and microcirculation are also considered important effects in acupoint activation and acupuncture mechanisms [27]. Previous results indicated the mean blood flow was larger at the acupoints than in their surrounding tissues, indicating when an acupoint was stimulated adequately, the blood perfusion of this point continued to increase and improve local microcirculation in specific organs and tissues [28, 29]. The immune cells, mast cells, may play a crucial role in mediating the establishment of networks among the circulatory, nervous and immune system at the acupoints [30]. Our results suggested after stimulation of the BL60 and KI3 in the AIA joint with laser, the TNF-α in the tibiotarsal joint reduced significantly, which is in accordance with our previous study[31]. This is probably because LA can promote local microcirculation to increase waste removal and adequate nutrient delivery to chondrocytes.

Although these results generally show potentially favorable outcomes with LA for enhancing cartilage repair in an AIA rat model, this study does have some limitations. The progression of RA is multifactorial, for example, many pathological factors including aggrecanases, matrix metalloproteinases and nitric oxide result in an imbalance between the anabolic and catabolic processes of ECM, finally inducing cartilage destruction [14]. Yet, the detailed molecular mechanisms of LA underlying cartilage damage in AIA are not fully examined in this study. In addition, degradation of subchondral bone may also be a main contributor to RA progression. Further investigations will focus on the effects of LA intervention on the breakdown of ECM and histopathological changes of subchondral bone in an AIA model.

Our experiment may provide some evidence or insights for understanding the molecular mechanism on the chondroprotective effects of LA in RA. Our study demonstrated LA benefits cartilage repair through enhancing an anabolic process of ECM by promoting ECM macromolecules, especially collagen type II, proteoglycans and COMP. Further experiments are necessary to reveal the chondroprotective mechanisms involving the catabolic processes of ECM and whether LA can suppress breakdown of ECM by matrix metalloproteinases to decrease cartilage destruction, as well as the specific compounds that can delay progressive joint space narrowing characteristic of arthritis and improve the biomechanics of articular joints by protecting chondrocytes.

## Conclusion

To our knowledge, this study is the first to present outcomes of LA on protection against cartilage pathohistological degradation in rats with AIA-induced cartilage damage. Our study’s results demonstrate LA intervention may be effective in delay of cartilage degradation and cartilage repair by promoting CoII, proteoglycans and COMP expressions in ECM, reducing inflammatory cell infiltration and TNF-α in cartilage. Whether catabolic processes of ECM can also be modulated by LA for reducing the cartilage destruction in RA deserves further intensive research.
